# Evaluation of the Association between Obesity Markers and Type 2 Diabetes: A Cohort Study Based on a Physical Examination Population

**DOI:** 10.1155/2021/6503339

**Published:** 2021-12-28

**Authors:** Tengfei Yang, Bo Zhao, Dongmei Pei

**Affiliations:** ^1^Department of Health Management, Shengjing Hospital of China Medical University, Shenyang, China; ^2^Department of Pulmonary and Critical Care Medicine, Shengjing Hospital of China Medical University, Shenyang, China

## Abstract

**Purpose:**

To evaluate the predictive effect of different obesity markers on the risk of developing type 2 diabetes in a population of healthy individuals who underwent physical examination and to provide a reference for the early detection of individuals at risk of diabetes.

**Methods:**

This retrospective cohort study included 15206 healthy subjects who underwent a physical examination (8307 men and 6899 women). Information on the study population was obtained from the Dryad Digital Repository. Cox proportional risk models were used to calculate the hazard ratio (HR) and 95% confidence interval (CI) of different obesity markers, including the lipid accumulation index (LAP), body mass index (BMI), waist-to-height ratio (WHtR), visceral adiposity index (VAI), and body roundness index (BRI) on the development of type 2 diabetes. The effectiveness of each obesity marker in predicting the risk of developing type 2 diabetes was analyzed using the receiver operating characteristic curve (ROC curve) and the area under the curve (AUC).

**Results:**

After a mean follow-up of 5.4 years, there were 372 new cases of type 2 diabetes. After correcting for confounding factors such as age, sex, smoking, alcohol consumption, exercise, and blood pressure, Cox proportional risk model analysis showed that elevations in BMI, LAP, WHtR, VAI, and BRI increased the risk of developing type 2 diabetes. The ROC curve results showed that LAP was the best predictor of the risk of developing diabetes, with an AUC (95% CI) of 0.759 (0.752–0.766), an optimal cutoff value of 16.04, a sensitivity of 0.72, and a specificity of 0.69.

**Conclusion:**

An increase in the BMI, LAP, WHtR, VAI, and BRI can increase the risk of developing type 2 diabetes, with LAP being the best predictor of this risk.

## 1. Introduction

Diabetes is a commonly occurring disease in humans and is a serious health threat. The 2017 International Diabetes Federation statistics show that there are approximately 425 million adults with diabetes worldwide, and low- and middle-income countries bear almost 80% of the diabetes burden. China has emerged as the country with the largest diabetic population in the world, with 114 million people aged 20–79 years in the country suffering from the disease [1]. In the face of health and social problems caused by diabetes, early detection and intervention are the main methods of diagnosis and treatment [2]. Previous studies have shown an independent positive relationship between obesity and type 2 diabetes [3] high level of obesity might increase the risk of developing diabetes [4, 5]. Therefore, selection of appropriate obesity markers can help detect and better prevent and control the onset and progression of diabetes.

Many obesity markers have been reported, with their advantages and limitations. In the past, the most common marker for assessing obesity was the body mass index (BMI), which can be used to measure body weight; however, it cannot effectively distinguish between the fat and muscle [6]. Waist circumference (WC), waist-to-height ratio (WHtR), and waist-hip ratio (WHR) are recognized markers for evaluating central obesity in China and other countries and can reflect the degree of abdominal fat accumulation; however, they cannot accurately reflect visceral fat associated with metabolic disorders [7]. Compared with the above traditional obesity evaluation indices, the lipid accumulation index (LAP) [8], visceral fat index (VAI) [9], and body roundness index (BRI) [10] are new body mass indices that have been proposed in recent years, which are important in reflecting the degree of lipid accumulation and visceral fat content in the human body [11]. Among them, the lipid accumulation product (LAP) combines the measurement of WC and triglycerides (TG) and can better measure the degree of visceral fat accumulation. Several studies have shown that the LAP is closely associated with cardiovascular disease [12], diabetes [13], and stroke [14].

Currently, studies on the association between different anthropometric markers of obesity and the development of type 2 diabetes are mainly cross-sectional studies [15, 16], and there is a paucity of large cohort studies. Therefore, this study is the first to analyze the association between five anthropometric markers of obesity (BMI, WHtR, LAP, VAI, and BRI) and the risk of developing type 2 diabetes based on the data of mature cohort studies in the Dryad database. In addition, the predictive validity of the new obesity markers was compared with those of traditional obesity markers for the risk of developing type 2 diabetes. This study will provide a scientific basis for the early diagnosis of type 2 diabetes.

## 2. Subjects and Methods

### 2.1. Study Subjects

Data of 15206 patients with T2DM were extracted from the Dryad Digital Repository (10.5061/dryad.8q0p192), which was shared by Okamura et al. [17]. The database contained the physical examination results of patients who underwent physical examination at the Murakami Memorial Hospital (MMRH) between 2004 and 2015 and did not have diabetes at their first physical examination. The primary endpoint of the study was set as type 2 diabetes mellitus. In addition, subjects with a baseline fasting glucose ≥ 6.1 mmol/L were excluded from the database [18] because the proportional risk could not be assumed. As all data were obtained from an online database, institutional ethics approval was not necessary.

### 2.2. Baseline Data Collection

The clinical information extracted included age; sex; BMI; weight; WC; history of smoking; history of alcohol consumption; history of exercise; levels of triglycerides, total cholesterol, high-density lipoprotein (HDL), aspartate transaminase (AST), alanine transaminase (ALT), and gamma-glutamyl transpeptidase (GGT); systolic blood pressure (SBP); diastolic blood pressure (DBP); follow-up duration; and the occurrence of type 2 diabetes. The diagnostic criteria for the development of type 2 diabetes were HbA1c ≥ 6.5% and fasting blood glucose ≥ 7.0 mmol/L [19]. Of these, 258 cases were excluded as their WC data did not meet the criteria for LAP calculation.

### 2.3. Formula for Calculating the Obesity Index

The following are the formulas used for calculating the obesity index:

1. Body mass index (BMI) = weight (kg)/height^2^ (m^2^) [20]
2. Lipid accumulation index (LAP): LAP (male) = [WC − 65] × TG and LAP (female) = [WC − 58] × TG, where the WC unit is cm [21]
3. Waist − to − height ratio (WHtR) = waist circumference/height [20]
4. Visceral adiposity index (VAI): VAI (male) = {waist circumference (cm)/[39.68 + (1.88 × body mass index)]} × (triglycerides (mmol/L)/1.03) × (1.31/HDL (mmol/L)) and VAI (female) = {waist circumference (cm)/[36.58 + (1.89 × body mass index)]} × (triglycerides (mmol/L)/0.81) × (1.52/HDL (mmol/L)) [22]
5. Body roundness index (BRI): BRI = 364.2 − 365.5 × {1 − [(waist circumference (m)/2*π*)/(0.5 × height (m))]^2^}^1/2^ [23]

The subjects were divided into groups Q1, Q2, Q3, and Q4 based on the quartiles of BMI, LAP, WHtR, VAI, and BRI.

### 2.4. Statistical Processing

SPSS version 22.0 (IBM Corp., Armonk, NY) was used for data processing and analysis. As the data did not follow a normal distribution, quantitative data were expressed as the median and interquartile range, and the Mann–Whitney *U* test was used to compare the two groups. Qualitative data were expressed as frequencies and percentages, and the *χ*^2^ test was used for comparison between groups. The Cox proportional risk model was used to calculate the hazard ratio (HR) and 95% confidence interval (CI) to explore the association between different obesity markers and the risk of developing type 2 diabetes. MedCalc statistics was used to plot ROC curves to evaluate the predictive effect of each obesity marker on type 2 diabetes. The Delong test was used to compare the differences in AUC between different groups, and *P* < 0.05 was considered to be statistically significant.

## 3. Results

### 3.1. Basic Information of Study Subjects

A total of 15206 individuals were enrolled in this study. The mean follow-up period was 5.4 years, and the number of new cases of type 2 diabetes during the follow-up period was 372, with a cumulative incidence of type 2 diabetes of 2.5%. The difference between the proportions of subjects who exercised between the T2DM and non-T2DM groups was not statistically significant (*P* > 0.05). Age, smoking rate, alcohol consumption rate, BMI, LAP, WHtR, VAI, BRI, SBP, DBP, AST, ALT, GGT, TC, TG, and HDL-C levels were higher in the T2DM group than in the non-T2DM group, and all differences were statistically significant (*P* < 0.01). Specific data are shown in Table 1.

### 3.2. ROC Curve Analysis of Different Markers for Predicting T2DM

**Figure 1:**
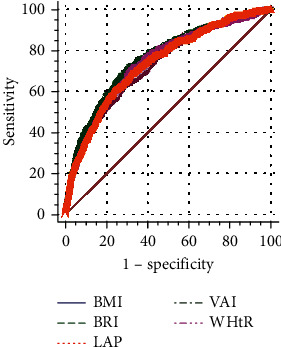
ROC curves of five obesity evaluation markers for predicting the onset of type 2 diabetes.

The results of ROC analysis and AUCs with their corresponding 95% CI parameters are shown in Figure 1 and Table 2. The test variables were BMI, LAP, WHtR, VAI, and BRI, and the status variable was type 2 diabetes. The maximum AUC value was for LAP [AUC = 0.759, 95% CI (0.752–0.766)], and the difference between the AUC values for LAP and other test variables was statistically significant when the Delong test was used for two-way comparisons.

### 3.3. Comparison of the Association between Different Obesity Markers and the Development of Type 2 Diabetes

**Figure 2:**
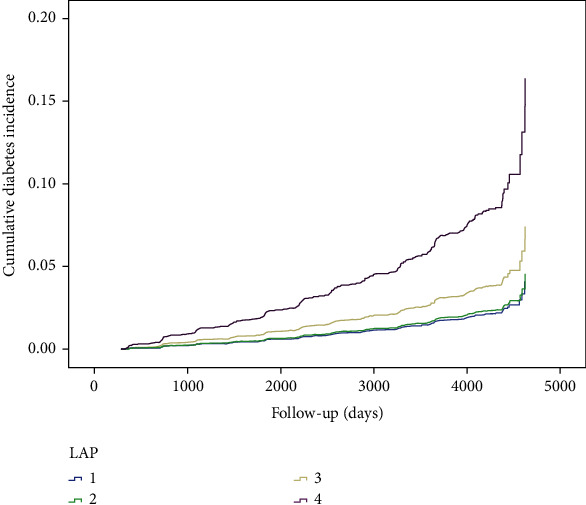
Cumulative prevalence of type 2 diabetes mellitus (T2DM) according to the lipid accumulation index (LAP) quartile groups.

An analysis of the association between the five obesity evaluation markers and new-onset T2DM is shown in Table 3. The risk of developing T2DM increased with an increase in the LAP, BMI, WHtR, VAI, and BRI. After further correction for age, sex, smoking, alcohol consumption, and other factors, as well as T2D, the above associations decreased but remained statistically significant. In corrected model 3, the risk (HR) and 95% CI for T2D development at the highest quartile (Q4) compared with its lowest quartile (Q1) for each of the five obesity evaluation markers were LAP: 3.94 (2.55–6.09), VAI: 2.85 (1.91–4.25), BMI: 3.87 (2.49–6.01), BRI: 4.5 (2.93–6.91), and WHtR: 4.5 (2.93–6.91). The trends in different subgroups of LAP and the cumulative incidence of type 2 diabetes are shown in Figure 2. There was no statistically significant difference between the second quartile and first quartile of LAP, BMI, WHtR, VAI, and BRI for risk (HR) of T2D.

## 4. Discussion

The present cohort study compared different anthropometric measures of obesity (LAP, BRI, BMI, WHtR, and VAI) for their ability to identify type 2 diabetes in generally healthy subjects who underwent physical examination. Our results suggest that an increase in the mean LAP, BRI, BMI, WHtR, and VAI can increase the risk of developing type 2 diabetes after adjusting for potential confounders. Further analysis showed that LAP was the strongest predictor for developing type 2 diabetes; BMI had the poorest predictive power compared with other body fat markers. This suggests that obesity markers such as LAP could be reliable predictors of type 2 diabetes. Community general physicians could screen at-risk populations using anthropometric markers of obesity and provide targeted interventions for the prevention and control of type 2 diabetes.

The idea that obesity increases the risk of developing metabolic diseases such as type 2 diabetes, and cardiovascular disease is widely recognized [24, 25]. Obesity markers are related to the level of reactive oxygen species. Excessive production of reactive oxygen species can cause insulin resistance [26]. In addition, the increase of inflammatory markers (endocan) in adipocytes is associated with the impairment of insulin signaling pathway [22, 27]. The BMI is the most widely used marker of obesity [28]; it was the first marker to be proposed and used for this purpose. However, it does not accurately reflect the distribution of body fat and cannot distinguish between fat and muscle. Studies have shown that the effect of obesity on blood glucose is not only associated with the body fat content but also associated with the accumulation sites of body fat [29, 30]. When compared to the BMI, the WHtR can better assess abdominal obesity. The results of this study showed that the incidence of diabetes mellitus in the subject population increased gradually with an increase in BMI and WHtR, and the results of further Cox proportional risk model calculations revealed that the BMI and the WHtR are independent risk factors for diabetes mellitus; the risk (HR) and 95% CI were 4.5 (2.93–6.91) and 3.87 (2.49–6.01), respectively. Analyses of the ROC curve showed an AUC = 0.74 and 95% CI of 0.733–0.747 for the WHtR, which was higher than the AUC = 0.730 and 95% CI of 0.723–0.737 for the BMI, and the difference between the predictive values of the BMI and the WHtR for diabetes was found to be statistically different. A meta-analysis [31] showed that the WHtR was slightly more accurate than the BMI and the WHR in predicting diabetes, which is consistent with the results of this study. Another study [32] using a multistage randomized whole-group sampling method that recruited 5000 subjects from a representative community sample in Sri Lanka pointed out that the best cutoff point for WHtR screening for diabetes was 0.517, which is close to the cutoff point of 0.496 reported in this paper.

Compared to traditional obesity anthropometric markers, new obesity anthropometric markers can further differentiate between abdominal wall fat (abdominal subcutaneous fat) and intra-abdominal fat (visceral fat), which was shown in previous studies to have a greater impact on metabolic diseases than abdominal wall fat [33]. WC and WHtR do not differentiate between these two types of fat [34]. The VAI and the BRI, which are new markers of obesity, are good markers of visceral adiposity and insulin sensitivity [9, 35] and show good correlation and predictive ability for diabetes, cardiovascular disease, and hypertension [36–38]. However, the results of the present study showed no significant difference in the AUC values of VAI, BRI, and WHRT, and their abilities to predict the onset of diabetes mellitus were similar. Several studies have shown that the VAI has the strongest correlation with diabetes and insulin sensitivity than other anthropometric markers (BMI, WC, and WHR) [39]. The results of a 15-year cohort study by Wang et al. were in line with ours and concluded that the VAI independently predicted diabetes in the Chinese study population, although its predictive power was not superior to that of the BMI and the WC [40]. The inconsistent results may be due to differences in factors such as sample size and population characteristics.

The lipid accumulation index combines two markers of anatomy and physiology, the WC reflecting trunk fat content (including the amount of visceral fat) and the circulating TG content under fasting [8]. This is a new marker of obesity that could be used to complement existing commonly used markers. A cross-sectional study of 215,651 Chinese adults found that the LAP was more accurate than the BMI in predicting type 2 diabetes [41]. In this study, after adjusting for the influencing factors of age, sex, SBP, and DBP using the Cox proportional risk model analysis, the risk of developing type 2 diabetes was 3.94 times higher for subjects in the LAPQ4 (fourth quartile) group than for those in the LAPQ1 (first quartile) group and 1.78 times higher for those in the LAPQ3 group than for those in the LAPQ1 group, and these differences were statistically significant. The area under the ROC curve was further used to analyze the ability of LAP and four different obesity markers (BMI, WHtR, BRI, and VAI) in predicting the development of type 2 diabetes. The results showed that LAP had the largest AUC = 0.759 and 95% CI of 0.752–0.766 and was significantly different from the other four markers in a pairwise comparison. It is suggested that the strongest association of LAP with the development of type 2 diabetes might be because the LAP is closely associated with the degree of insulin resistance [42] and important aspect in the pathogenesis of diabetes. When the storage and buffering capacity of adipose tissue are exceeded, excess lipids are stored ectopically in nonadipose tissues, such as the liver, kidney, and pancreatic B cells, leading to lipotoxicity, which causes insulin resistance and impairment of islet cell function, ultimately leading to the development of diabetes [43, 44].

In addition, the present study has certain limitations. The publicly available cohort database lacks insulin level measurements and cannot be analyzed for insulin resistance. Therefore, we could only interpret the results according to existing pathophysiological models and were unable to perform an in-depth exploration of the pathogenic mechanisms of LAP. In addition, the relationship between LAP and cerebrocardiovascular disease complications of type 2 diabetes was not investigated; more self-constructed prospective cohort studies are required for verifying our findings.

In conclusion, body measurement markers are closely associated with the development of T2DM in a population of healthy people who underwent physical examination, and increased levels of BMI, WHtR, LAP, VAI, and BRI can increase the risk of developing type 2 diabetes, with the LAP being the best predictor of the risk of developing diabetes. We recommend that general physicians should identify individuals with LAP elevation in community health screening and control LAP levels through prompt diet modification and exercise to prevent the occurrence of T2DM, to the maximum extent possible.

## Figures and Tables

**Table 1:** Comparison of baseline characteristics between the T2DM and non-T2DM groups.

Variables	Non-T2DM cases (*n* = 14834)	Incident T2DM cases (*n* = 372)	*Z*/*X*^2^	*P*
Age	42 (37-50)	46 (41-52.75)	-7.53	*P* < 0.01
Sex				
Men	8022 (54.08%)	87 (23.39%)	137.34	*P* < 0.01
Women	6812 (45.92%)	285 (76.61%)		
Smoke				
No	8713 (58.73%)	144 (38.71%)	111.21	*P* < 0.01
Yes	6121 (41.27%)	288 (61.29%)		
Alcohol consumption				
No	11321 (76.32%)	266 (71.50%)	4.64	0.03
Yes	3512 (23.68%)	106 (28.50%)		
Sports				
No	12221 (82.39%)	321 (86.29%)	3.83	0.05
Yes	2613 (17.61%)	51 (13.71%)		
SBP (mmHg)	113 (103.5-124)	121.5 (112-132)	-9.52	*P* < 0.01
DBP (mmHg)	71 (64-78)	76.5 (70-84)	-10.29	*P* < 0.01
ALT (U/L)	17 (13-23)	24 (18-39)	-14.82	*P* < 0.01
AST (U/L)	17 (14-21)	20 (16-26)	-9.13	*P* < 0.01
GGT (U/L)	15 (11-22)	24 (17-36.75)	-13.68	*P* < 0.01
TG (mmol/L)	0.73 (0.5-1.11)	1.22 (0.86-1.93)	-14.87	*P* < 0.01
TC (mmol/L)	5.07 (4.5-5.66)	5.43 (4.81-6)	-6.80	*P* < 0.01
HDL-C (mmol/L)	1.42 (1.17-1.71)	1.13 (0.96-1.32)	-14.43	*P* < 0.01
Anthropometric indices				
BMI	21.8 (19.96-23.89)	24.67 (22.29-27.25)	-15.18	*P* < 0.01
VAI	0.73 (0.46-1.24)	1.52 (0.92-2.53)	-15.84	*P* < 0.01
BRI	2.64 (2.11-3.24)	3.52 (2.85-4.29)	-15.83	*P* < 0.01
LAP	9.77 (4.88-19.18)	26.9 (14.33-47.07)	-17.09	*P* < 0.01
WHtR	0.46 (0.43-0.49)	0.51 (0.47-0.55)	-15.83	*P* < 0.01

**Table 2:** ROC curve results of five obesity evaluation markers for predicting new-onset type 2 diabetes.

	AUC (95% CI)	Cutoff	Sensitivity (%)	Specificity (%)	*Z*	*P*
VAI	0.740 (0.733-0.747)	1.044	0.718	0.677	2.471	0.014
BRI	0.740 (0.733-0.747)	3.289	0.605	0.766	2.047	0.041
BMI	0.730 (0.723-0.737)	23.521	0.629	0.713	3.035	0.002
LAP	0.759 (0.752-0.766)	16.044	0.723	0.689		
WHtR	0.740 (0.733-0.747)	0.496	0.605	0.766	2.047	0.041

BRI vs. BMI: *Z* = 1.445, *P* = 0.148; BRI vs. VAI: *Z* = 0.007, *P* = 0.994; BMI vs. VAI: *Z* = 0.682, *P* = 0.495; WHtR vs. BMI: *Z* = 1.445, *P* = 0.148; WHtR vs. BRI: *Z* = 0, *P* = 1; WHtR vs. VAI: *Z* = 0.007, *P* = 0.994.

**Table 3:** Results of Cox proportional risk regression model analysis of the relationship between different obesity markers and the development of T2DM.

	New cases/total (*n*)	Incidence density (%)	Model 1	Model 2	Model 3
LAP					
Q1 (<4.97)	27/3821	0.71	1	1	1
Q2 (4.97-10)	36/3796	0.95	1.39 (0.85-2.29)^∗^	1.2 (0.73-1.99)^∗^	1.09 (0.66-1.81)^∗^
Q3 (10-19.76)	76/3795	2	2.91 (1.88-4.51)	2.23 (1.42-3.48)	1.78 (1.13-2.81)
Q4 (>19.76)	233/3794	6.14	8.56 (5.75-12.75)	6.1 (4.02-9.25)	3.94 (2.55-6.09)
*P* for linear trend			<0.001	<0.001	<0.001
VAI					
Q1 (<0.46)	30/3799	0.79	1	1	1
Q2 (0.46-0.74)	34/3799	0.89	0.97 (0.59-1.58)^∗^	0.86 (0.52-1.4)^∗^	0.78 (0.48-1.27)^∗^
Q3 (0.74-0.1.26)	88/3799	2.32	2.44 (1.61-3.7)	1.94 (1.28-2.95)	1.59 (1.04-2.42)
Q4 (>1.26)	220/3798	5.79	6 (4.1-8.79)	4.23 (2.86-6.26)	2.85 (1.91-4.25)
*P* for linear trend			<0.001	<0.001	<0.001
BRI					
Q1 (<2.12)	27/3802	0.71	1	1	1
Q2 (2.12-2.65)	44/3801	1.16	1.79 (1.11-2.9)^∗^	1.55 (0.96-2.51)^∗^	1.43 (0.88-2.32)^∗^
Q3 (2.65-3.26)	75/3802	1.97	2.99 (1.93-4.64)	2.26 (1.44-3.53)	1.8 (1.14-2.83)
Q4 (>3.26)	226/3801	5.95	9.59 (6.43-14.29)	6.77 (4.49-10.2)	4.5 (2.93-6.91)
*P* for linear trend			<0.001	<0.001	<0.001
BMI					
Q1 (<19.99)	26/3803	0.68	1	1	1
Q2 (19.99-21.86)	50/3800	1.32	1.87 (1.16-3)^∗^	1.59 (0.99-2.56)^∗^	1.44 (0.89-2.32)^∗^
Q3 (21.86-23.97)	87/3802	2.29	3.22 (2.08-4.99)	2.42 (1.55-3.79)	2 (1.27-3.15)
Q4 (>23.97)	209/3801	5.5	7.93 (5.28-11.92)	5.92 (3.89-9.01)	3.87 (2.49-6.01)
*P* for linear trend					
WHtR					
Q1 (<0.42)	27/3802	0.71	1	1	1
Q2 (0.42-0.46)	44/3801	1.16	1.79 (1.11-2.9)^∗^	1.55 (0.96-2.51)^∗^	1.43 (0.88-2.32)^∗^
Q3 (0.46-0.49)	75/3802	1.97	2.99 (1.93-4.64)	2.26 (1.44-3.53)	1.8 (1.14-2.83)
Q4 (>0.49)	226/3801	5.95	9.59 (6.43-14.29)	6.77 (4.49-10.2)	4.5 (2.93-6.91)
*P* for linear trend			<0.001	<0.001	<0.001

Model 1: unadjusted; Model 2: adjusted for age, sex, smoke, and alcohol; Model 3: adjusted for model 2 plus ALT, AST, GGT, SBP, and DBP. ^∗^*P* > 0.05.

## Data Availability

Extra data can be accessed via the Dryad data repository at 10.5061/dryad.8q0p192.

## Additional Points

*Code Availability*. Code availability is not applicable.
